# *Notes from the Field*: Fatal Yellow Fever in a Traveler Returning From Peru — New York, 2016

**DOI:** 10.15585/mmwr.mm6634a5

**Published:** 2017-09-01

**Authors:** Alexandra P. Newman, Rebecca Becraft, Amy B. Dean, Rene Hull, Bryon Backenson, Gillian Hale, Janeen Laven, Julu Bhatnagar, J. Erin Staples

**Affiliations:** ^1^New York State Department of Health; ^2^Chemung County Health Department, Elmira, New York; ^3^Wadsworth Center, New York State Department of Health; ^4^Division of High-Consequence Pathogens and Pathology, National Center for Emerging and Zoonotic Infectious Diseases, CDC; ^5^Division of Vector-borne Diseases, National Center for Emerging and Zoonotic Infectious Diseases, CDC, Ft. Collins, Colorado.

In October 2016, a male New York resident aged 74 years developed fever, myalgia, nausea, and vomiting while traveling in Peru, 3 days after visiting the northern Amazon area. During the next 2 days, he experienced fever, abdominal pain, and watery diarrhea and was admitted to a hospital in Peru, where *Entamoeba histolytica* was detected in his stool. He was treated with intravenous fluids and antibiotics and released 1 day after admission. His condition worsened, however, and he returned to New York and immediately sought care at a hospital emergency department, where he was found to be afebrile, slightly confused, and jaundiced. Laboratory tests revealed leukopenia, thrombocytopenia, acute renal failure, liver dysfunction, and a metabolic acidosis (Table). He was transferred from the emergency department to a tertiary care center, where he was admitted and received intravenous fluids, antibiotics, and hemodialysis. During the next 2 days, he developed melena and disseminated intravascular coagulation. He experienced multiple episodes of ventricular fibrillation and died 3 days after admission. Autopsy revealed gastrointestinal hemorrhage and subtotal hepatocellular necrosis. Testing for selected viral, bacterial, and parasitic agents was negative, except for antibody to Salmonella H type A/B (Table). He had not received yellow fever vaccine before traveling. Serum specimens and tissues were sent to Wadsworth Center, the New York State Public Health Laboratory, and CDC to test for yellow fever virus and other pathogens.

**TABLE:** Clinical laboratory results* and infectious disease test results for patient with a fatal case of yellow fever — New York, 2016

Laboratory test	Result	Reference range
White blood cell count	3,600^†^	3,800–10,600/*μ*l
Platelet count	5,300^†^	150,000–400,000/*μ*l
Bicarbonate	10^†^	22–303 mmol/L
Sodium	135	134–145 mmol/L
Potassium	5.7^†^	3.5–5.1 mmol/L
Blood urea nitrogen	151^†^	9–20 mg/dL
Creatinine	13.7^†^	0.8–1.5 mg/dL
Alanine amino transferase	3,584^†^	21–72 U/L
Aspartate amino transferase	3,596^†^	17–59 U/L
Total bilirubin	11.8^†^	0.0–1.0 mg/dL
Alkaline phosphatase	349^†^	38–126 U/L
Albumin	3.4	3.5–5.0 g/dL
Lactic acid	3.6^†^	0.7–2.1 mmol/L
Bacterial cultures (blood)	No growth	No growth
Leptospiral DNA (urine)	Not detected	Not detected
Dengue viral RNA (serum)	Not detected	Not detected
Salmonella H type A/B antibodies (serum)	Positive^†^	Negative
Q fever antibodies (serum)	Negative	Negative
Hepatitis A virus antibodies (serum)	Nonreactive	Nonreactive
Hepatitis B virus antibodies (serum)	Nonreactive	Nonreactive
Hepatitis C virus antibodies (serum)	Nonreactive	Nonreactive
Yellow fever virus immunoglobulin M antibodies	Positive^†^	Negative
Yellow fever virus neutralizing antibodies	640^†^	<10

* Upon hospital admission.

^†^ Outside the reference range.

A serum specimen collected 7 days after illness onset tested positive for flaviviral RNA by reverse transcription–polymerase chain reaction (RT-PCR), and the amplicon sequencing was consistent with yellow fever virus. A serum specimen obtained at autopsy was positive for yellow fever immunoglobulin M antibodies. Yellow fever RT-PCR assays performed on RNA extracted from formalin-fixed, paraffin-embedded liver tissue were positive; amplicon sequence analysis revealed highest identity with wild-type yellow fever virus strains. An immunohistochemical assay for yellow fever virus performed on the liver tissue demonstrated staining of necrotic hepatocytes throughout the lobules, without mesenchymal staining. The morphologic features of fulminant active hepatitis and the immunohistochemical staining pattern and sequencing results, in combination with the patient’s travel history to a region of Peru where yellow fever is endemic, lack of yellow fever vaccination, and clinical history supported the diagnosis of infection with wild-type yellow fever virus (*1*).

Yellow fever is a mosquitoborne viral disease endemic to sub-Saharan Africa and tropical areas of South America. Most infections are asymptomatic or result in a nonspecific febrile illness. The severe form of yellow fever results in jaundice and hemorrhage; approximately 50% of severe cases are fatal (*2*).

During 1970–2015, 11 yellow fever cases were reported among U.S. and European travelers (*3*). Before the current case, the last yellow fever case diagnosed in a U.S. resident was in 2002 (*4*). However, after large outbreaks in Africa and South America during 2016–2017, the number of cases confirmed in travelers from countries without endemic yellow fever transmission increased substantially, including at least 11 workers infected in Angola; two travelers in Peru; one each in Suriname and Bolivia; and this case (*5*,*6*).

No specific treatment for yellow fever exists; care is based on symptoms. Prevention of infection is through vaccination and avoidance of mosquito bites. Yellow fever vaccine is recommended for persons aged ≥9 months who are traveling to or living in areas at risk for yellow fever virus transmission (*3*). However, because serious adverse events can occur after yellow fever vaccination, contraindications and precautions to vaccination, such as patient age, should be considered before administering the vaccine. Health care providers should consider and test for yellow fever in unvaccinated persons with fever and jaundice or hemorrhage who live in or have traveled to an area with yellow fever virus transmission. Information on current yellow fever outbreaks and vaccination requirements and recommendations for specific countries are available on CDC Travelers' Health website (https://www.cdc.gov/travel/).
